# Supplementary material for: A viral glycoprotein targets IgG+ memory B cells to mediate humoral immune evasion
Source: EMBO Mol Med. 2026 Jan 20;18(2):795–823. doi: 10.1038/s44321-026-00372-1 (PMC12905349; doi:10.1038/s44321-026-00372-1)
Supplement: Supplementary file 1 — Appendix [file 44321_2026_372_MOESM1_ESM.pdf]

Appendix

A viral glycoprotein targets IgG+ memory B cells to mediate humoral immune evasion

Precious Cramer[1,2,3,#,$], Stefan F.H. Neys[4,#], Manuela Fiedler[5,6], Raquel Lorenzetti[4], Henrike Reinhard[5], Iga Janowska[4], Julian Staniek[4], Ann-Katrin Kohl[1], Petra Hadlova[7,8,], Magdalena Huber[1], Bodo Plachter[9], Clarissa Read[10,11], Valeria Falcone[1], Jens von Einem[9], Katja Hoffmann[1], Tihana Lenac Rovis[12], Stipan Jonjic[12], Philipp Kolb[1], Marta Rizzi[4,7,13,14,§,*], and Hartmut Hengel[1,§,*]

[1]Institute of Virology, Medical Center and Faculty of Medicine, University of Freiburg. 79104 Freiburg, Germany
[2]Spemann Graduate School of Biology and Medicine (SGBM), University of Freiburg. 79104 Freiburg Germany.
[3]Faculty of Biology, University of Freiburg. 79104 Freiburg Germany
[4]Department of Rheumatology and Clinical Immunology, Medical Center and Faculty of Medicine, University of Freiburg 79104 Freiburg, Germany
[5]Institute of Virology, Heinrich-Heine-University, University Hospital of Düsseldorf, Universitätsstr. 1, 40225 Düsseldorf, Germany
[6]Berlin Institute of Health (BIH) at Charité - Universitätsmedizin Berlin, Berlin, Germany
[7]Division of Clinical and Experimental Immunology, Institute of Immunology, Center for Pathophysiology, Infectiology and Immunology, Medical University of Vienna, Vienna, Austria.
[8]CLIP-Cytometry, Department of Paediatric Hematology and Oncology, 2nd Medical School, Charles University, V Uvalu 84, 150 06 Prague 5, Czech Republic
[9]Institute for Virology, University Medical Center of the Johannes Gutenberg-University Mainz, D-55131 Mainz, Germany
[10]Institute of Virology, Ulm University Medical Center, 89081 Ulm, Germany.
[11]Central Facility for Electron Microscopy, Ulm University, 89081 Ulm, Germany.
[12]Center for Proteomics University of Rijeka Faculty of Medicine Brace Branchetta 20, 51000 Rijeka, Croatia.
[13]Center for Chronic Immunodeficiency, University Medical Center Freiburg, Faculty of Medicine, University of Freiburg, Freiburg, Germany.
[14]CIBSS – Centre for Integrative Biological Signalling Studies, University of Freiburg, Freiburg, Germany.

[#]Shared first author.
[§]Equal contribution and shared senior authorship

[$]Present address: Center for Virology and Vaccine Research, Beth Israel Deaconess Medical Center, Harvard Medical School, 3 Blackfan Circle Boston MA 02215.

*Corresponding authors:
hartmut.hengel@uniklinik-freiburg.de; Tel.: +49-761-203-6533; ORCID: 0000-0002-3482-816X

marta.rizzi@uniklinik-freiburg.de; Tel.: +49-761-270-62170; ORCID: 0000-0002-5153-60089

Table of contents:

Appendix Fig S1 A-C. Interaction of $gp34_{1-179}$ with lymphocytes 3

Appendix Fig S2 A-D. The IgG+ BCR mediates selective binding of $gp34_{1-179}$ to B cells 4

Appendix Fig S3 A-B. Receptor ligation at distinct binding domains of the BCR show unaltered proximal signalling events and comparable total protein levels 5

Appendix Fig S4 A-C. $gp34_{1-179}$ inhibits B cell proliferation and plasmablast formation 6

Appendix Fig S5. TNF-α neutralization does not affect the gp341-179-induced halt in B cell proliferation and differentiation 7

Appendix Fig S6. Pre-incubation of IgG with $gp34_{1-179}$ blocks binding of CD32b to immune complexes (CD20/Rtx) 8

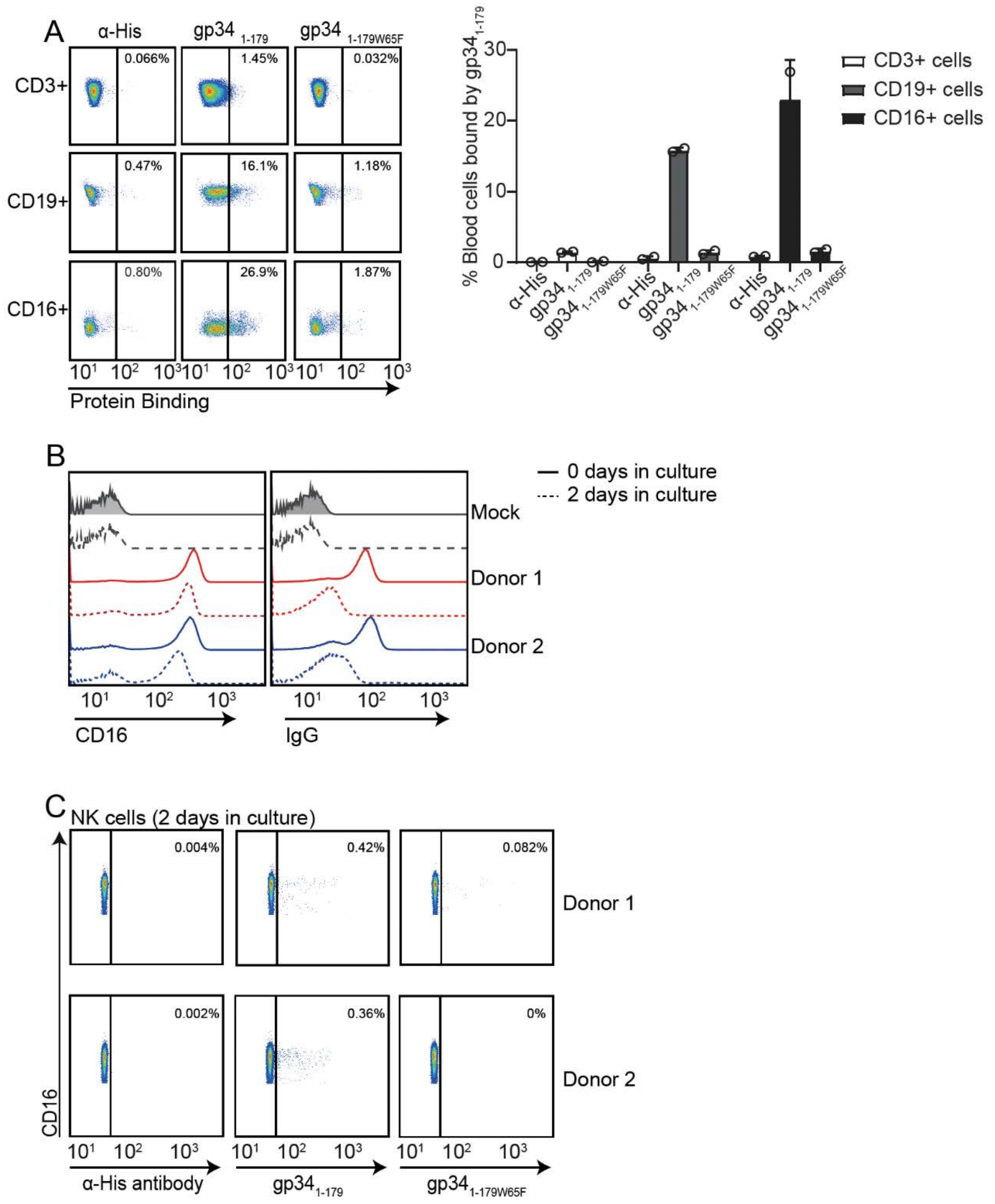

Appendix Fig S1. Interaction of gp34$_{1-179}$ with lymphocytes.

(A) Isolated PBMCs incubated with gp34$_{1-179}$ and gp34$_{1-179W65F}$ were stained with fluorophore-conjugated antibodies (CD3-FITC, CD16-BV421, and CD19-PerCP). Lymphocyte populations to which the recombinant proteins bound were identified in flow cytometry with an α-His-PE antibody. Bar graphs are the percentages of cells bound by each recombinant protein in the total lymphocyte population. Each circle represents two independent experiments. Error bars =SD. **(B)** Primary NK cells isolated from two donors were cultured for 2 days and stained for surface expression of CD16 and cytophilic IgG by flow cytometry. **(C)** gp34$_{1-179}$ and gp34$_{1-179W65F}$ were tested for binding to CD16^{+} NK cells cultured for 2 days in flow cytometry.

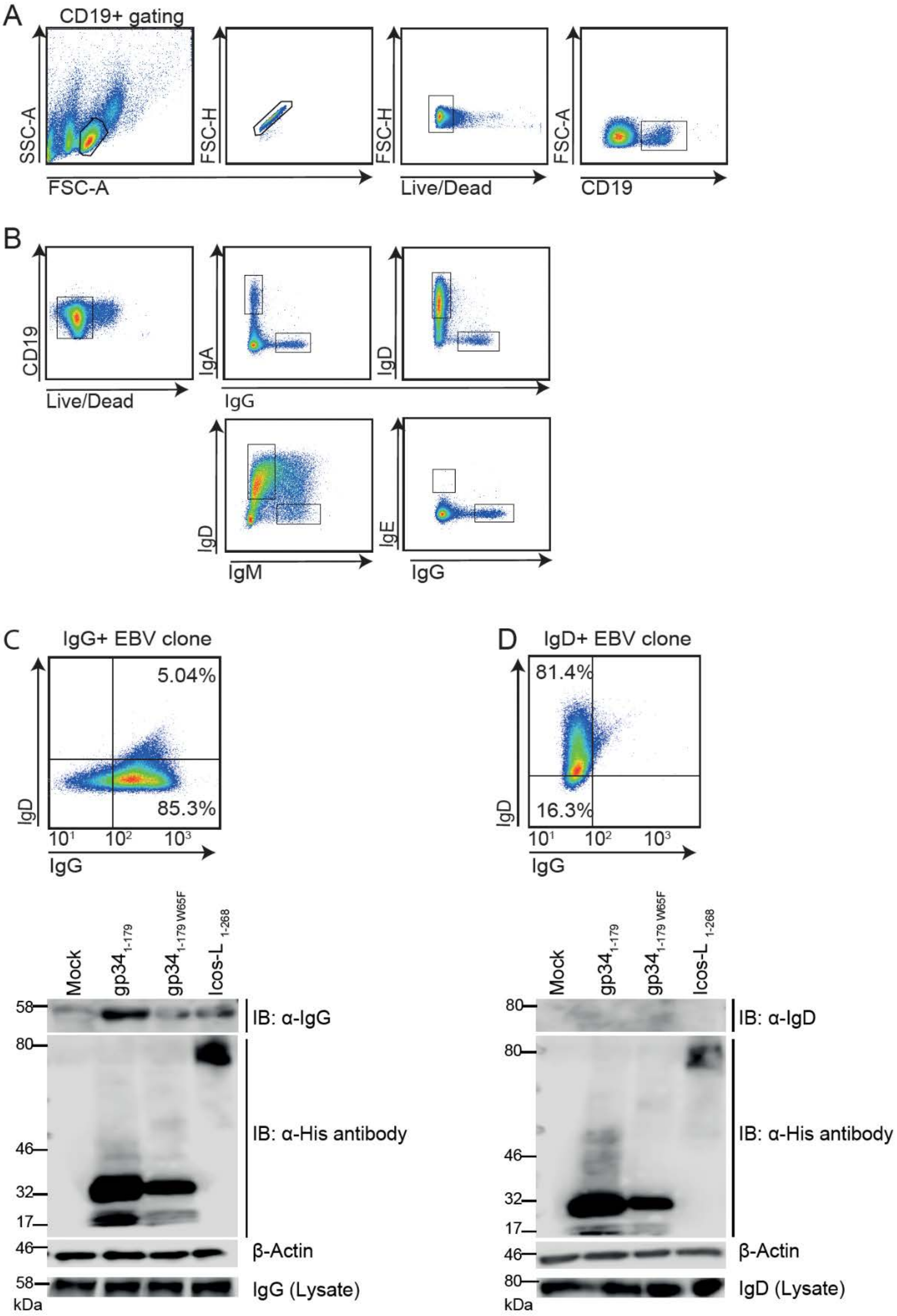

Appendix Fig S2. The IgG+ BCR mediates selective binding of gp34$_{1-179}$ to B cells.

(A) Back gating identifying live CD19^{+} B cells in the PBMC population and **(B)** Characterization of B cell isotypes in flow cytometry using surface specific antibodies. **(C)** Surface expression of IgG BCR and **(D)** IgD BCR on Epstein-Barr virus (EBV) transformed B cells was analyzed in flow cytometry. Histidine tagged gp34$_{1-179}$, gp34$_{1-179W65F}$ and ICOS-L$_{1-268}$ coupled to histidine resins were incubated with lysates from the IgG BCR or IgD BCR B cell clones. Co-precipitated complexes were eluted and separated on a 10% SDS-PAGE. α-His antibody was used to probe for the bound recombinant proteins to the resin. α-IgG and α-IgD antibodies were used in analyzing co-precipitated IgG^{+} BCR and IgD^{+} BCR with the respective recombinant proteins.

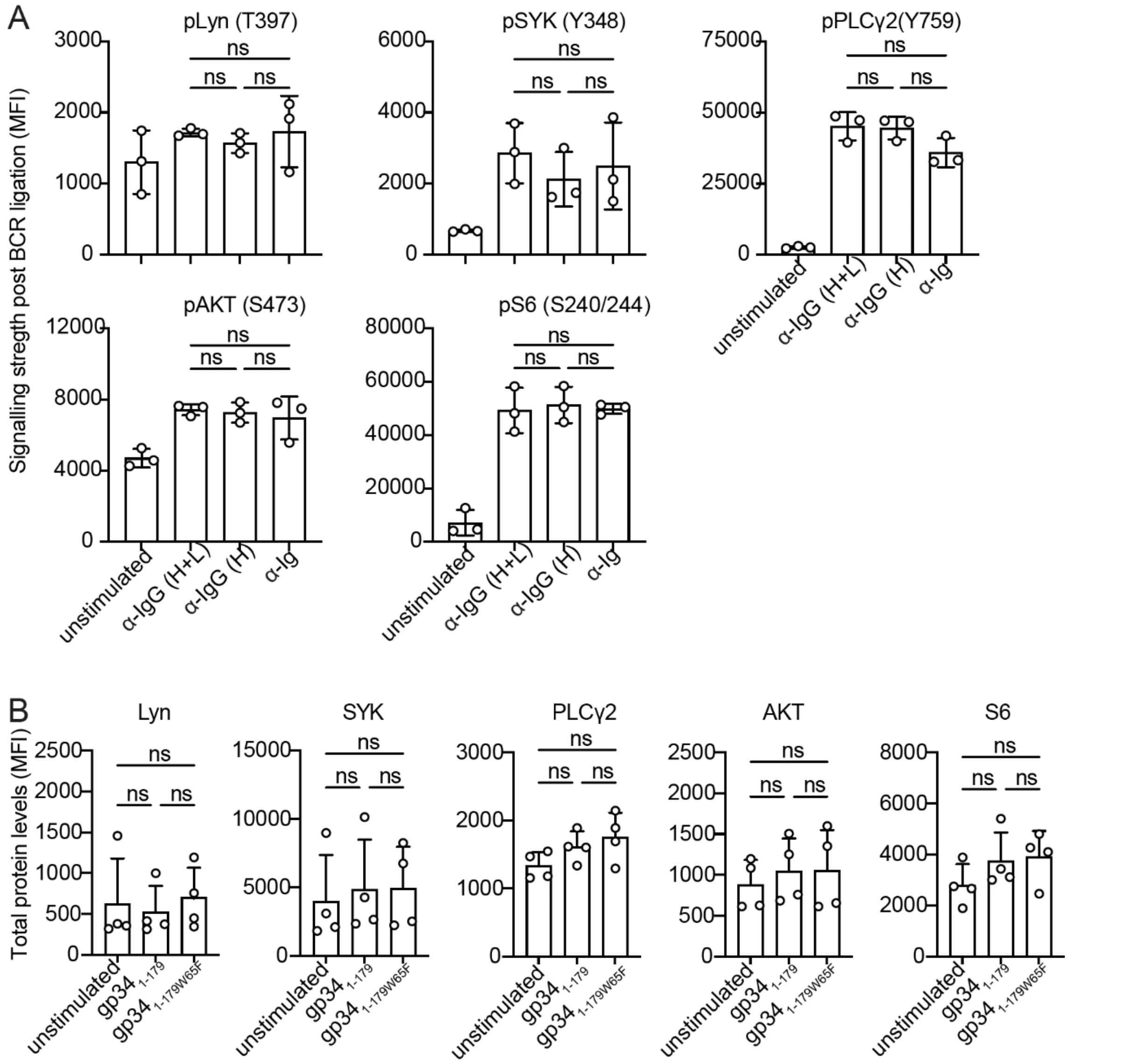

Appendix Fig S3. Receptor ligation at distinct binding domains of the BCR show unaltered proximal signalling events and comparable total protein levels. **(A)** IgG+ B cells were stimulated through BCR dimerization via heavy and light chains (α-IgG (H+L)), heavy chain only (α-IgG (H)), or total Ig (α-Ig). Phosphorylation of downstream BCR signalling molecules were measured after 10 minutes (p-Lyn, p-SYK, and p-PLCγ2) or 1 hr (p-AKT and p-S6) post incubation with the indicated stimuli. Data are three biological replicates which were analyzed by Kruskal-Wallis test. Error bars = Mean ± SD **(B)** Total B cells from four independent patients (n =4) were incubated with gp341-179, gp341-179W65F, or left unstimulated. Following fixation and permeabilization, the total protein levels of Lyn, SYK, PLCγ2, AKT and S6 were determined by flow cytometry. Differences between groups were analyzed using a Friedmann test, and error bars the Standard deviation of the Mean.

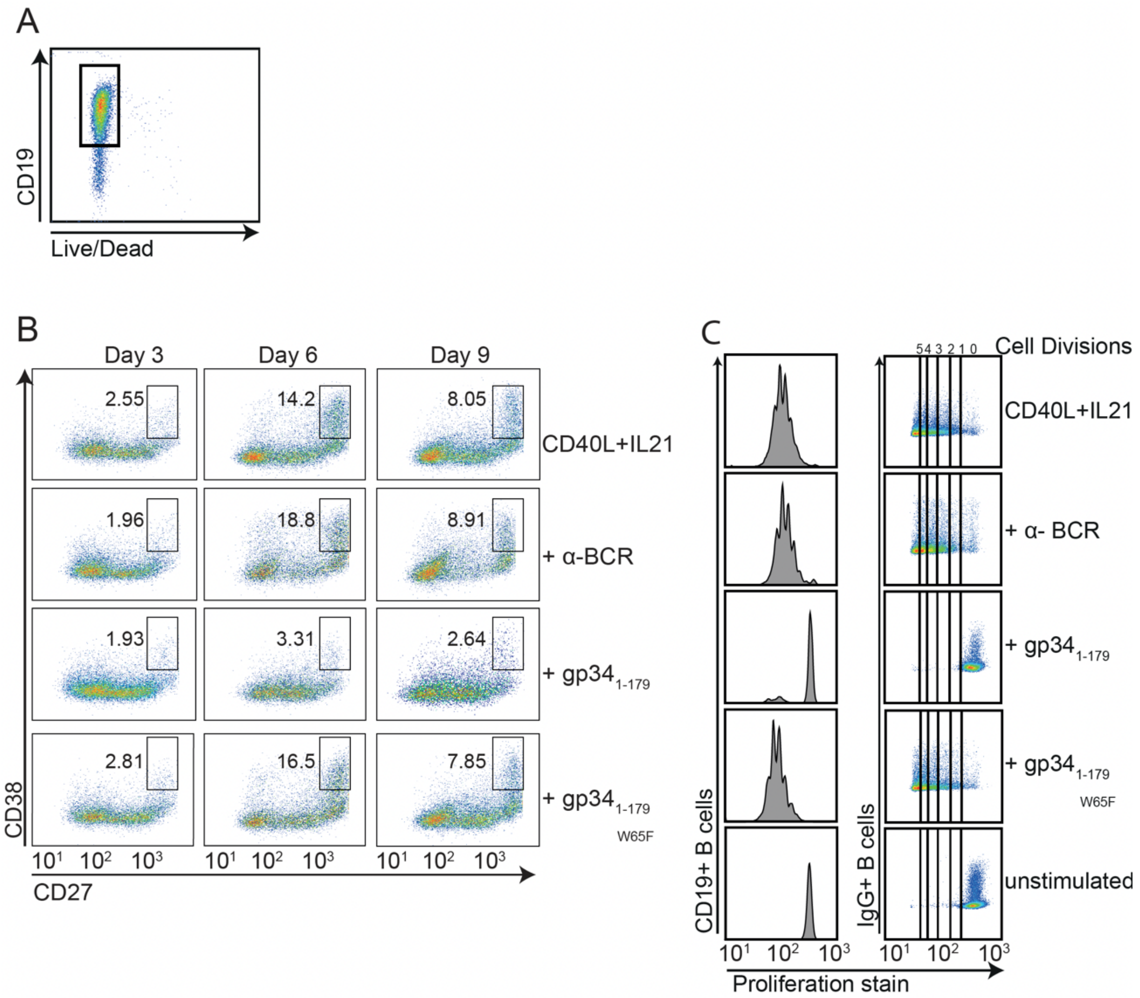

Appendix Fig S4. gp34$_{1-179}$ inhibits B cell proliferation and plasmablast formation.
(A) Gating of live CD19+ B cells and **(B)** plasmablasts (CD38^{++}CD27^{++}) in *in vitro* cultured B cells treated with CD40L/IL-21 in the presence of gp34$_{1-179}$, gp34$_{1-179W65F}$, or α-BCR for 3, 6, and 9 days. Numbers in each plot represent the percentage of plasmablasts formed on a given day. **(C)** Primary B cells were pre-stained with cell trace violet and stimulated with gp34$_{1-179}$, gp34$_{1-179W65F}$, or α-BCR in the presence of CD40L/IL-21 for 6 days. Proliferation of B cells in each condition was analyzed by flow cytometry. Data is a representation of two independent experiments.

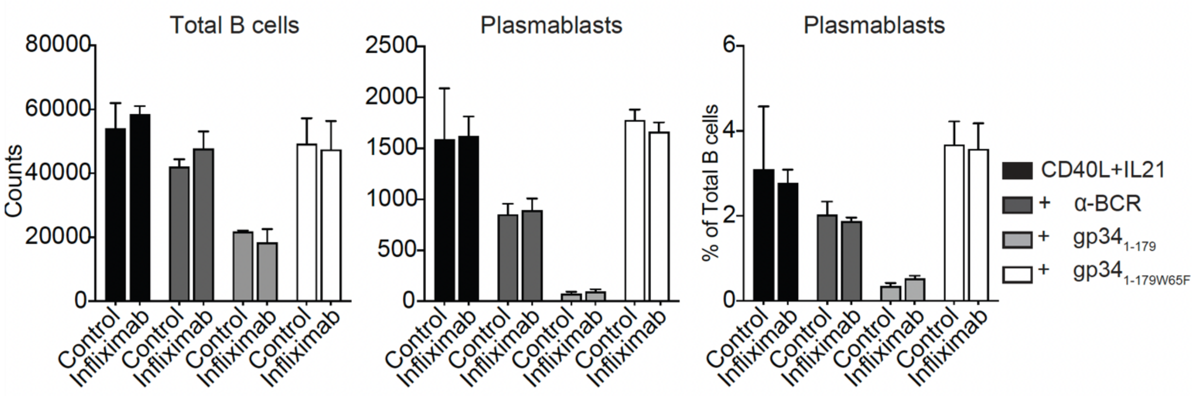

Appendix Fig S5. TNF-α neutralization does not affect the gp341-179-induced halt in B cell proliferation and differentiation. Total B cells were cultured with the indicated stimuli in the presence of 1 µg/ml infliximab or control F(ab')2 fragments. Total B cell counts, and plasmablast counts and frequencies were assessed by flow cytometry after 3 and 6 days (shown for day 3). Data was generated from 3 independent experiments and error bars = Mean ± SD.

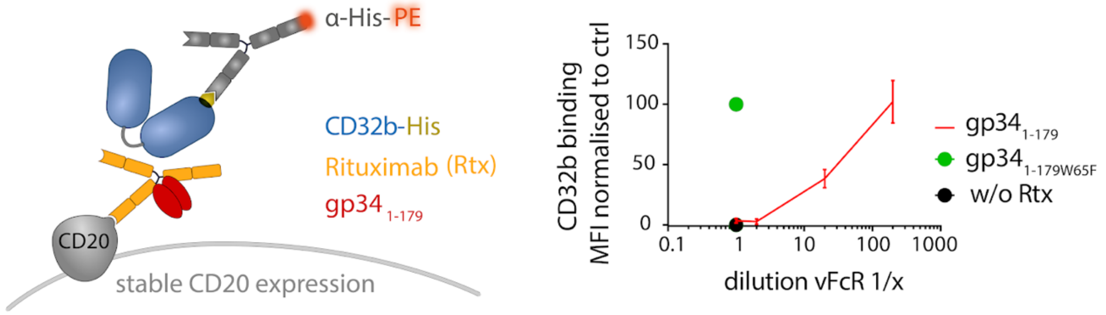

Appendix Fig S6. Pre-incubation of IgG with gp34$_{1\text{-}179}$ blocks binding of CD32b to immune complexes (CD20/Rtx).

293T cells stably expressing CD20 were incubated with preformed Rtx/gp34$_{1\text{-}179}$ or Rtx/gp34$_{1\text{-}179W65F}$ complexes (1hr, 4° C) and detected with FcγR/α-His-PE in flow cytometry. Data are three independent experiments performed in duplicates. Error bars = SD.
