# Supplementary material for: A viral glycoprotein targets IgG+ memory B cells to mediate humoral immune evasion
Source: EMBO Mol Med. 2026 Jan 20;18(2):795–823. doi: 10.1038/s44321-026-00372-1 (PMC12905349; doi:10.1038/s44321-026-00372-1)
Supplement: Supplementary file 3 — Expanded View Figures [file 44321_2026_372_MOESM3_ESM.pdf]

Expanded View Figures

Figure EV1. Functional characterization of truncated gp34 variants and ICOS ligand.

(**A**) The predicted structure of gp34 (Atalay et al, 2002) includes an N terminal signal sequence (S) of 23 amino acids (aa 1–23), a connecting Ig-like domain (aa 24–122) with 3 N-linked glycosylation sites (Y) and the putative intramolecular disulfide bridge (dashed bracket), a 22 aa transmembrane domain (TM; aa 183-204), and a C terminal cytoplasmic tail of 30 aa in length (aa 205–234). Truncation variants of gp34 were cloned and tested for Fcγ binding properties. The truncation variants of gp34 were expressed by rVACV for 14 h in CV-1 cells. ^{35}S metabolic labeling of cells for 1 h was followed by precipitation of proteins from lysates with human IgG-Fc fragment. Half of the precipitate from each sample was deglycosylated with Endoglycosidase H overnight at 37 °C. Separation of proteins was performed by 10–13% gradient SDS–PAGE. (**B**) The tryptophan-to-phenylalanine substitution at position 65 of the $gp34_{1\text{-}179}$ sequence designated as $gp34_{1\text{-}179W65F}$ variant was used as a non-Fcγ binding control. Both variants were expressed in CV-1 cells for 14 h using rVACV. Metabolic labeling was followed by precipitation of the proteins from the lysates with human IgG-Fc fragment. Proteins were separated by 12% SDS–PAGE. Immunoprecipitation with α-FLAG-coupled agarose served as an expression control. (**C**) $gp34_{1\text{-}179}$, $gp34_{1\text{-}179W65F}$ and N terminal variant of ICOS ligand ($ICOS\text{-}L_{1\text{-}268}$) with V5 and His6 epitope tags were expressed in HEK293T cells. The supernatants were collected after 6 days and purified by affinity chromatography on the Äkta purification system. Maturation and glycosylation pattern of purified recombinant proteins was analyzed by digesting the proteins with EndoH and PNGase F overnight. Proteins were separated by 10% SDS–PAGE and detected with a peroxidase conjugated anti-His antibody. (**D**) Human Fcγ fragment was coated onto an ELISA plate and incubated with titrated amounts of purified $gp34_{1\text{-}179}$, $gp34_{1\text{-}179W65F}$ or $ICOS\text{-}L_{1\text{-}268}$. Peroxidase-conjugated anti-His antibody was used to detect bound proteins to the Fcγ fragment by spectrophotometry. (**E**) Skov3 cells expressing the Her2 antigen were incubated with Herceptin together with $gp34_{1\text{-}179}$, $gp34_{1\text{-}179W65F}$ or $ICOS\text{-}L_{1\text{-}268}$. Inhibition of Fcγ receptor activation was measured as mIL-2 production of BW5147-FcγRIII reporter cells. Mock condition represents the absence of reporter cells. The Bar graphs show two independent experiments performed in triplicates. Error bars =SD.

© The Author(s)

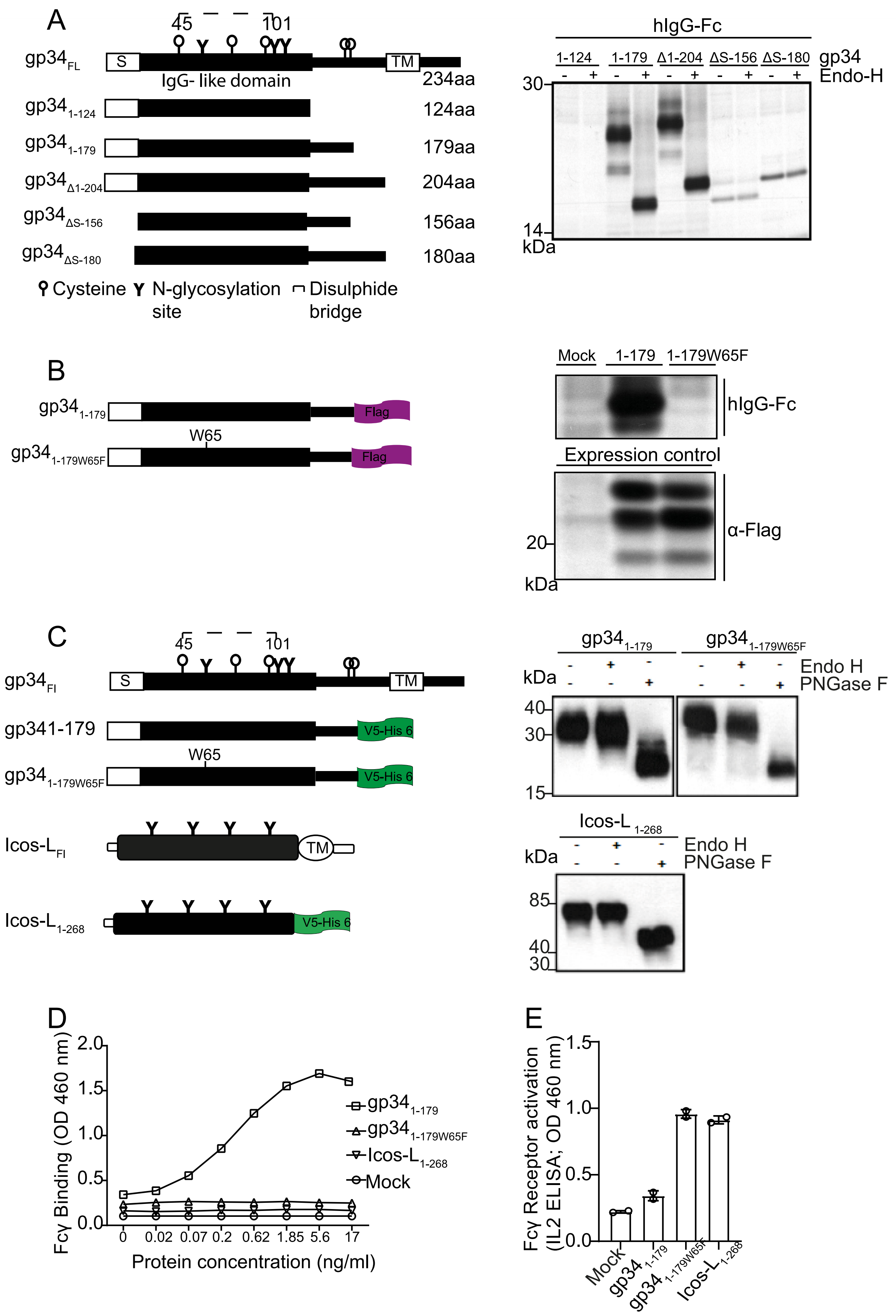
A
45
101
gp34FL
S
TM
IgG- like domain
234aa
gp34 1-124
124aa
gp34 1-179
179aa
gp34 Δ1-204
204aa
gp34 ΔS-156
156aa
gp34 ΔS-180
180aa
Cysteine
N-glycosylation site
Disulphide bridge
hIgG-Fc
1-124
1-179
Δ1-204
ΔS-156
ΔS-180
gp34
Endo-H
30
14
kDa
B
gp34 1-179
Flag
W65
gp34 1-179W65F
Mock
1-179
1-179W65F
hIgG-Fc
Expression control
α-Flag
20
kDa
C
gp34 Fl
gp341-179
V5-His 6
gp34 1-179W65F
Icos-L Fl
Icos-L 1-268
gp34 1-179
gp34 1-179W65F
Endo H
PNGase F
40
30
15
Icos-L 1-268
85
D
Fcγ Binding (OD 460 nm)
2.0
1.5
1.0
0.5
0.0
0
0.02
0.07
0.2
0.62
1.85
5.6
17
Protein concentration (ng/ml)
gp34 1-179
gp34 1-179W65F
Icos-L 1-268
Mock
E
Fcγ Receptor activation (IL2 ELISA; OD 460 nm)
Mock

 © The Author(s)

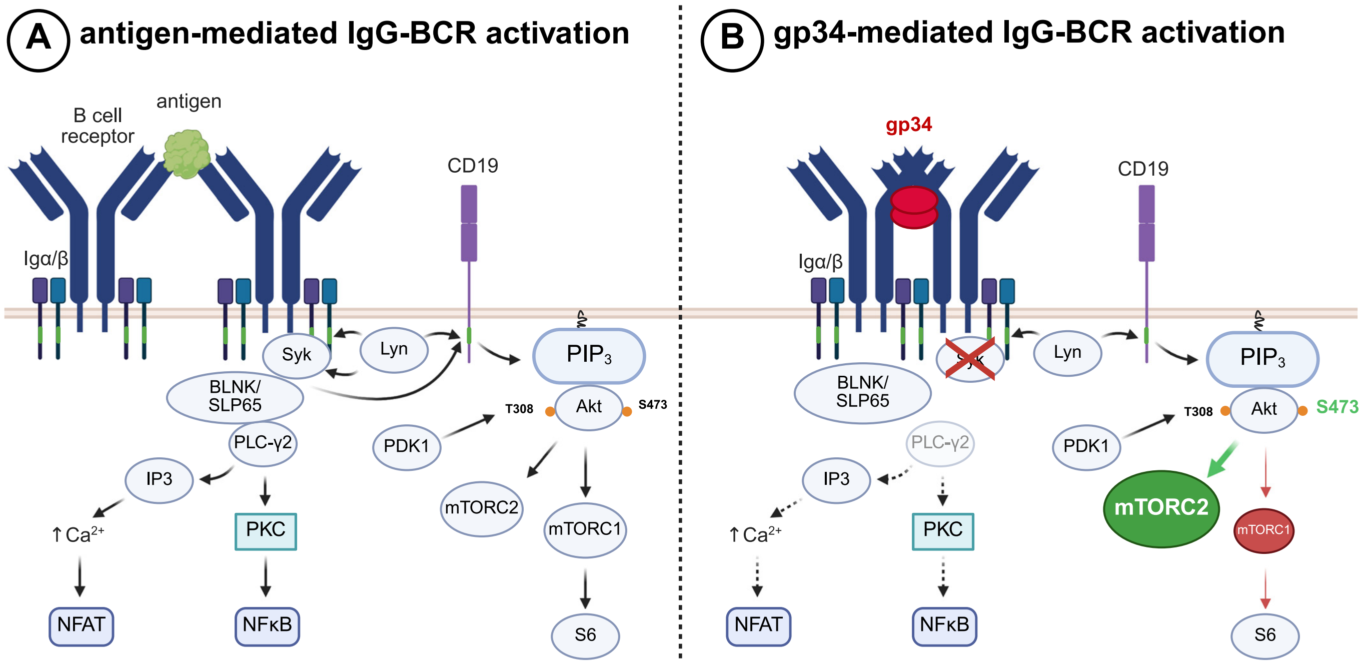

Figure EV2. IgG-BCR mediated signaling in memory B cells and its modification by gp34.

Model for (**A**) conventional antigen induced and (**B**) gp34 modified signaling pathways regulating memory B cell activation. Boxes indicate signaling effectors, ovals indicate signaling cascades. Transparency indicates lack of phosphorylation. Dashed arrows indicate downstream effects which were not experimentally addressed in this study. Adapted from Laidlaw and Cyster, Nat Rev Immunol. 21, 209–220 (2021); and Puri et al, Int Rev Immunol. 32:397–427 (2013).

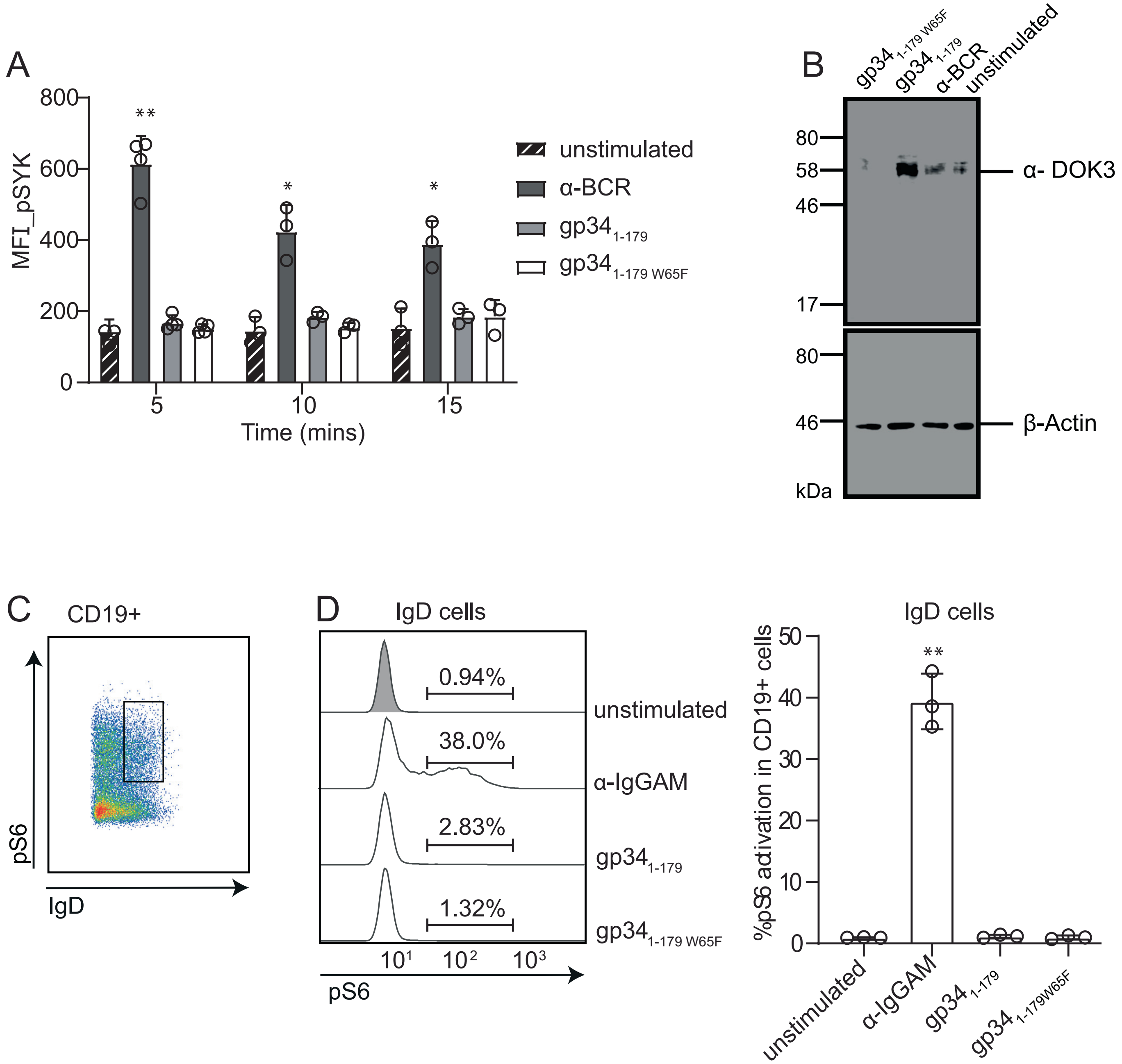

Figure EV3. gp34$_{1\text{-}17}$ blocks SYK activation, fails to induce S6 activation in IgD + B cells, yet upregulates DOK3 protein levels.

(**A**) Levels of phosphorylated SYK induced upon BCR stimulation with α-IgG, gp34$_{1\text{-}179}$ and gp34$_{1\text{-}179W65F}$ over the indicated time points, was examined by flow cytometry and depicted as MFI of three biological replicates. A two-way ANOVA (with mixed-effects analysis) was performed for statistical analysis, *$P = 0.0416$, **$P = 0.0388$. Data are shown as the mean ± standard of the mean. (**B**) B cells were stimulated with α-IgG, gp34$_{1\text{-}179}$, and gp34$_{1\text{-}179W65F}$ for 60 min at 37 °C. The cells were lysed and separated on a 12% SDS–PAGE. An α-DOK3 antibody was used to detect DOK3 expressed by B cells in each condition. Results are representative of two independent experiments. β-actin served as loading control. (**C**) Gating strategy showing IgD^{+} B cells expressing phosphorylated S6. (**D**) The histograms show the percentages of pS6^{+} cells in IgD^{+} B cells upon stimulation with gp34$_{1\text{-}179}$, gp34$_{1\text{-}179W65F}$ or α-BCR. Bar graphs depict frequency of pS6^{+} cells in IgD^{+} B cells in three biological replicates. A non-parametric Kruskal–Wallis test was performed to analyze the differences in each group (**$P = 0.0012$). Data are shown as the mean ± standard of the mean.

 © The Author(s)

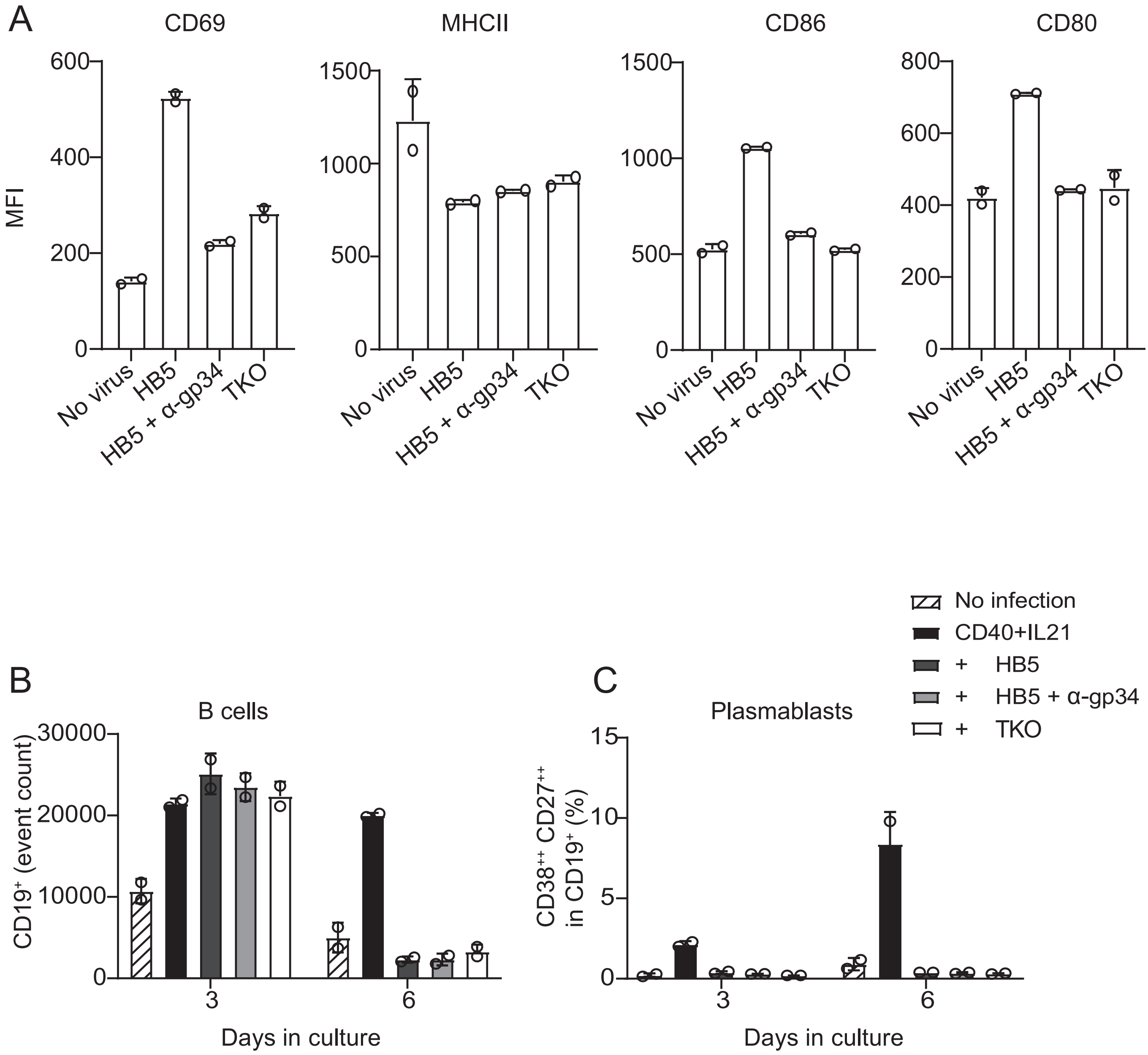

Figure EV4. HCMV virions activate B cells in a gp34 dependent manner.

(**A**) Primary B cells were incubated with HB5 or TKO virions in the presence or absence of α-gp34 mtrp.04 antibody. Upregulated CD69 and MHCII were examined after 24 h while CD86 and CD80 were assessed after 48 h by flow cytometry. (**B**, **C**) CD40L/IL21 stimulated primary B cells were incubated with HCMV HB5 virions and analyzed for CD19 B cell count and plasmablasts. All data are shown as the mean ± standard of the mean of two biological replicates.

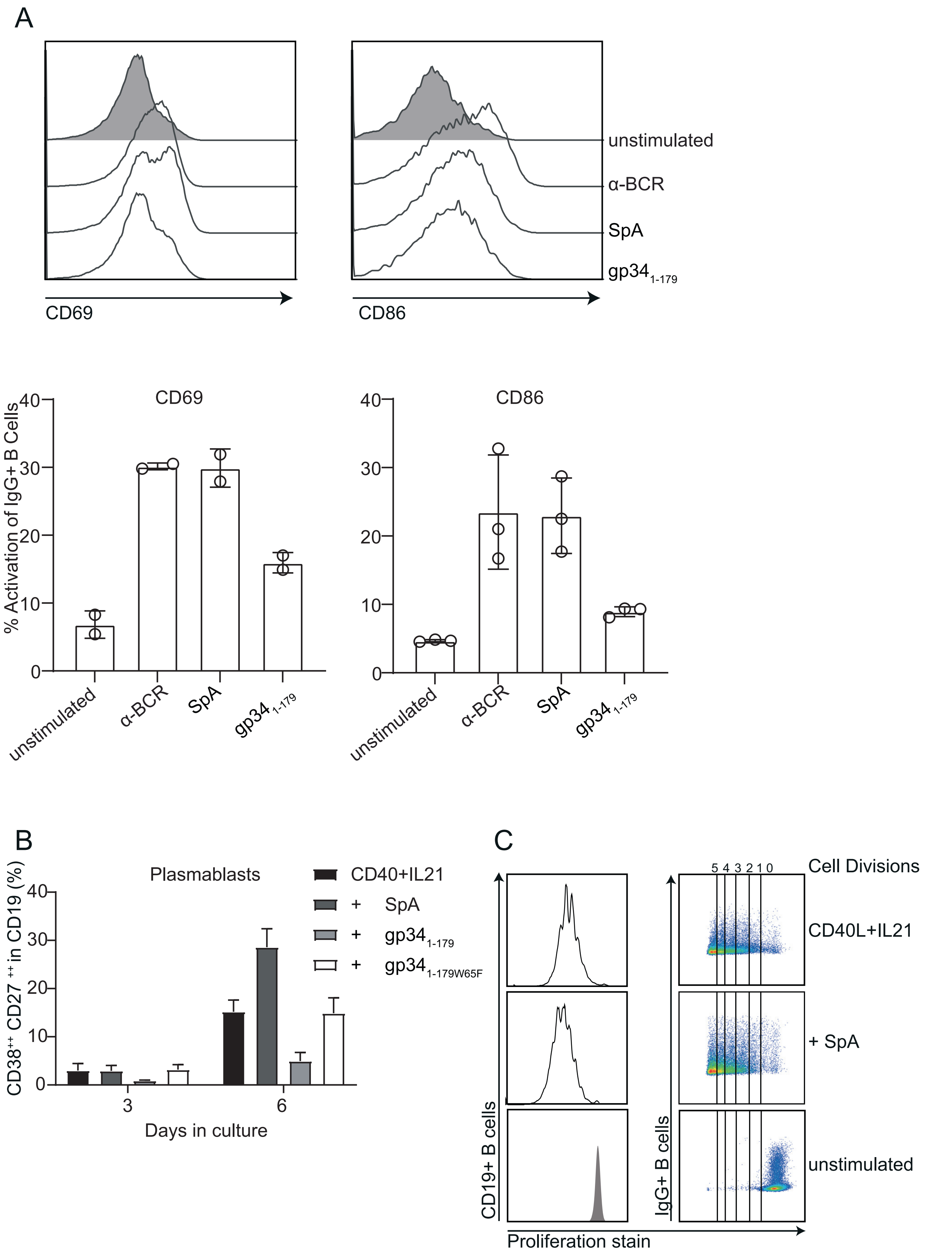
A
unstimulated
α-BCR
SpA
gp34 1-179
CD69
CD86
% Activation of IgG+ B Cells
B
Plasmablasts
CD40+IL21
+ SpA
+ gp34 1-179
+ gp34 1-179W65F
CD38++ CD27 ++ in CD19 (%)
Days in culture
C
Cell Divisions
5 4 3 2 1 0
CD40L+IL21
+ SpA
unstimulated
CD19+ B cells
IgG+ B cells
Proliferation stain

 © The Author(s)

Figure EV5. ***Staphylococcus aureus* protein A (SpA) activates IgG + B cells and supports proliferation and plasmablast formation.**

(**A**) Primary B cells were treated with 10 µg/mL each of α-BCR, SpA or $gp34_{1\text{-}179}$. Upregulation of activation markers CD69 and CD86 in IgG+ B cells were analyzed after 24 and 48 h respectively by flow cytometry. At least two biological replicates ($n = \geq$) were used in this experiment and error bars represent the standard deviation of the mean. (**B**) The CD19 + B cells were stimulated with 10 µg/mL of $gp34_{1\text{-}179}$, $gp34_{1\text{-}179W65F}$ or SpA in the presence of CD40L/IL21 for 3 and 6 days. The bars show the percentage of plasmablasts formed over the culture period. Data are represented as mean ± SEM of two independent experiments, each in duplicates. (**C**) B cells pre-stained with cell trace violet were treated with 10 ug/mL of SpA together with CD40L/IL-21, CD40L/IL-21 alone, or left unstimulated. Proliferation of the cells was examined after 6 days by flow cytometry.

© The Author(s)
